# RNAi-mediated inhibition of Lgr5 leads to decreased angiogenesis in gastric cancer

**DOI:** 10.18632/oncotarget.15770

**Published:** 2017-02-28

**Authors:** Hong-Qing Xi, Ke-Cheng Zhang, Ji-Yang Li, Jian-Xin Cui, Yun-He Gao, Bo Wei, Dongsheng Huang, Lin Chen

**Affiliations:** ^1^ Department of General Surgery, Chinese People's Liberation Army General Hospital, Beijing 100853, China; ^2^ Department of General Surgery, Zhejiang Provincial People's Hospital, Hangzhou 310014, China

**Keywords:** gastric cancer, Lgr5, angiogenesis, RNA interference

## Abstract

Leucine-rich repeat-containing G protein-coupled receptor 5 (Lgr5) is a novel gastric cancer marker. However, it is unclear whether it can play roles in tumor angiogenesis. In this study, we aim to investigate the role of Lgr5 on gastric cancer angiogenesis. Lgr5, VEGF expression levels and microvessel density (MVD) were detected in tumor tissue. Then, Lgr5 mRNA was downregulated by small interference RNA technique. Western blotting and real-time quantitative PCR (qRT-PCR) were performed to detect the expression of Lgr5 and VEGF protein and mRNA in Lgr5 siRNA-transfected gastric cancer cells. The effect of silencing Lgr5 on angiogenesis was examined by assessing human umbilical vein endothelia cell (HUVEC) capillary tube formation. The results indicated that Lgr5 expression was upregulated in gastric cancer and positively correlated with VEGF (r=0.305, P=0.001) and MVD (r=0.312, P=0.001). Silencing of Lgr5 expression resulted in suppression of VEGF mRNA and protein (all P=0.001). Moreover, when HUVECs were stimulated with conditioned medium from Lgr5 siRNA-transfected gastric cancer cells, tube formation was significantly decreased (2.51 ± 0.19 mm/mm^2^) compared with the treatment with regular cell culture medium (DMEM) (7.34 ± 0.30 mm/mm^2^) or medium from control siRNA-transfected cells (7.18 ± 0.33 mm/mm^2^) (all P=0.001). In conclusion, Lgr5 plays important roles in angiogenesis. Lgr5-specific siRNA could be designed into an effective therapeutic agent to inhibit gastric cancer angiogenesis.

## INTRODUCTION

Gastric cancer is among the most common malignant tumors and the second leading cause of cancer death worldwide [1]. Despite advances in diagnostic tools and therapeutic techniques, the 5-year survival rate is less than 30% [2] because of local invasion and metastasis, which is the main biological characteristic of gastric cancer [3]. Numerous studies have demonstrated that invasion and metastasis are highly dependent upon proliferation of tumor cells and angiogenesis, and thus antiangiogenic therapy could benefit cancer patients [4–7]. Therefore, searching for the molecular regulators of angiogenesis should be a major goal in gastric cancer research.

Leucine-rich repeat-containing G protein-coupled receptor 5 (Lgr5), also known as GPR49, FEX, GPR67, GRP49, and HG38, is a member of the G-protein-coupled receptor (GPCR) family and considered as a target of Wnt signaling [8–11]. Recent studies discovered that Lgr5 is a potential marker of adult stem cells of the small intestine, colon, stomach, and hair follicle bulge [12–14]. Lgr5 is overexpressed in several human tumors, including esophageal adenocarcinoma [15], gastric cancer [16, 17], colorectal cancer [18–20], hepatocellular carcinoma [10], ovarian cancer [21], and brain cancer [22]. Barker et al. [14] reported that Lgr5 appeared at the base of pyloric glands and could serve as a unique marker of stem cells in the stomach and transformation of adult Lgr5+ stem cells could lead to tumor formation in the stomach *in vivo*. Furthermore, Lgr5 also plays important roles in tumorigenesis and aggression and could be useful for the evaluation of clinical outcome of gastric cancer patients.

Angiogenesis is a complex event and requires the endothelial cell sprouting and tubule genesis, which is activated or inhibited by different transcription factors [23]. Microvessel density (MVD) is widely used for assessing angiogenesis in many human solid cancers [24–26].

Vascular endothelial growth factor (VEGF) and transforming growth factor-b1 (TGF-β1) [27] are key regulators of pathological and physiological angiogenesis [28, 29]. Elevated expression of VEGF has been reported in gastric cancer [30]. Wnt/β-catenin signaling can regulate vessel development in many aspects of angiogenesis, vascular remodeling and differentiation in pathological and physiological conditions [31, 32]. The central player of this pathway is the protein β-catenin [33]. β-catenin was also found to be overexpressed in gastric cancer [34], and accumulated in the cytosol and nucleus of proliferating vessels [35]. Previous studies have pointed out that activation of Wnt/β-catenin could promote vascular endothelial cell proliferation and survival by up-regulating the expression of VEGF [31, 36, 37]. Because of that there were seven β-catenin/TCF binding site in the promoter of VEGF, and β-catenin could combined with this site and then dramatically up-regulate levels of VEGF mRNA and protein [31].

As mentioned before, Lgr5, a member of the Wnt signaling complex at the membrane level, is considered as a target of Wnt signaling [8–11], and could active the Wnt/β-catenin signaling pathway [38]. Successful activation of the Wnt signaling pathway by Lgr5 could lead to phosphorylation of LRP receptors and eventually inhibit degradation of the crucial signaling molecule β-catenin. Accumulated β-catenin could then translocate to the nucleus to regulate the expression of target genes [11, 39], including VEGF. Therefore, we considered that Lgr5 participated in gastric cancer angiogenesis by enhancing Wnt/β-catenin signaling.

To our knowledge, there has been no investigation so far into the relationship between the expression of Lgr5 and tumor angiogenesis. We propose that Lgr5 could activate the expression and thereby affecting angiogenesis by activation of Wnt/β-catenin signaling. In present study, we first investigated the relationship between the expression of Lgr5 and VEGF and MVD status in gastric cancer tissue. We also observed the effect of Lgr5 on VEGF expression and angiogenesis *in vitro*.

## RESULTS

### Analysis of Lgr5 expression in gastric cancer and inter-relationships between Lgr5, VEGF, and MVD by Spearman's correlation test

**Figure 1:**
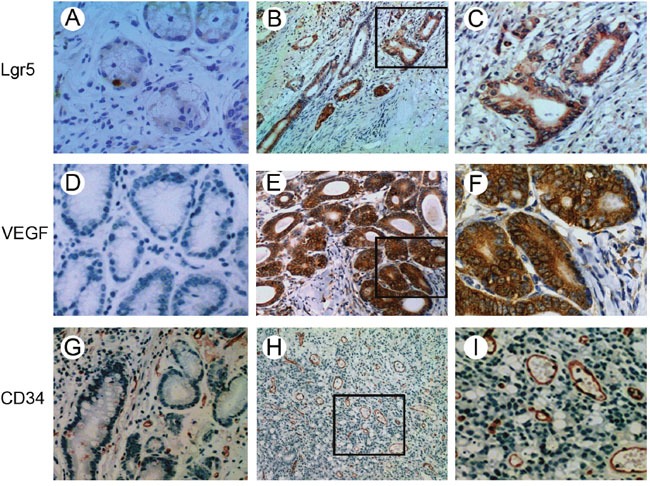
Immunohistochemical staining of Lgr5, VEGF and CD34 in gastric carcinoma and normal mucosa tissues **A**. Single epithelial cells were Lgr5-positive in normal mucosa tissues. **B** and **C**. Lgr5 staining in the cytoplasm of tumor cells. **D**. Lack of VEGF expression in normal mucosa tissues. **E** and **F**. VEGF was predominantly expressed in the cytoplasm. Microvessels were detected in normal mucosa tissues **G**. and gastric cancer tissues **H** and **I**. by immunohistochemical staining. (A, C, D, F, G and I: ×400 magnification; B, E and H: ×100 magnification).

Single epithelial cells were Lgr5-positive in normal mucosa tissue (Figure 1A). A diffuse and intense cytoplasmic staining pattern for Lgr5 was detected in gastric cancer tissue (Figure 1B and 1C). The positive rate of Lgr5 expression was 54.1% (172/318) in gastric cancer, which was much higher than that in the normal mucosal tissues (18.8%, 15/80, P=0.001). VEGF was seen in the cytoplasm. It was overexpressed in the gastric cancer tissues (65.1%, 207/318) and weakly expressed or absent in the normal mucosa (27.5%, 22/80) (Figure 1D, 1E, 1F).

The positive staining of CD34 was localized on the membrane of vascular endothelial cells (Figure 1G, 1H, 1I). Immunohistochemical staining of CD34 was used for MVD counting. The MVD of 318 tumor specimens ranged from 10 to 60 with a mean value of 30.11 ± 7.60. We chose a mean MVD value of 30 as the cut-off point for discrimination of the 318 patients and classified them into two subgroups: low MVD, MVD ≤ 30 and high MVD, MVD >30. A total of 128 cases (40.3%) were categorized as low MVD and 190 cases (59.7%) as high MVD (Figure 1H and 1I) (Table 1). There was a positive correlation between Lgr5 and VEGF expression in gastric cancer (P<0.001, r=0.305). A significant correlation between Lgr5 expression and MVD was also found (P<0.001, r=0.312).

**Table 1:** Correlations between Lgr5 and VEGF and MVD expression in gastric carcinoma

	Lgr5		*P*-value	*r* value
	Positive	Negative		
**VEGF expression**				
Positive	135	72	0.001	0.305
Negative	37	74		
**MVD**				
high MVD	127	63	0.001	0.312
low MVD	45	83		

*P*<0.05 was considered as statistically significant.

### Correlation between Lgr5 and VEGF protein relative expression by Pearson's correlation

**Figure 2:**
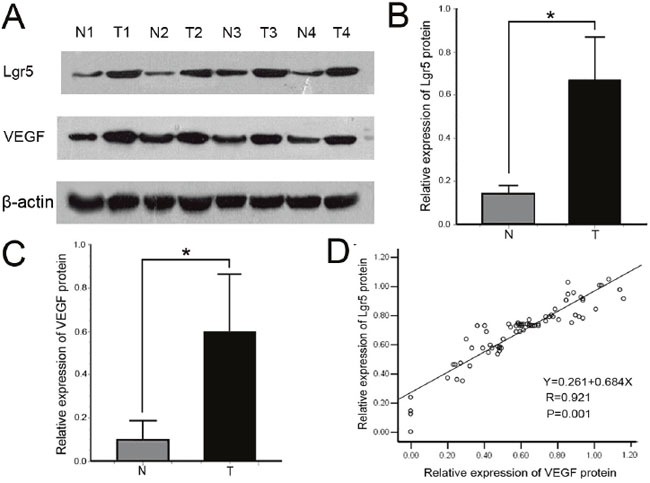
**A**. Representative immunoblots of Lgr5 and VEGF protein expression detected in whole tissue extracts from paired sample of gastric cancer tissue (T: tumor) and adjacent normal tissue (N: normal). β-actin was used as loading control. **B, C**. Comparison of Lgr5 and VEGF relative expression levels between gastric cancer tissue and adjacent normal tissue. Lgr5 and VEGF protein expression levels were higher in carcinoma than that in adjacent normal mucosa (all *P=0.001). Values are presented as the mean ± standard deviation (SD). **D**. Correlation between Lgr5 and VEGF protein expression levels in gastric cancer were analyzed by Pearson's correlation test and linear regression. Each protein level is relative to that of β-actin. There was a positive correlation between Lgr5 and VEGF (r = 0.921, *P = 0.001).

To evaluate the expression level of Lgr5 and VEGF protein, we performed western blot for Lgr5 and VEGF in 75 paired gastric cancer tissues (Figure 2). The mean relative expression of Lgr5 protein was 0.672±0.199 for cancer and 0.135±0.039 for adjacent normal mucosa. The mean relative expression of VEGF protein was (0.60±0.268) for cancer and (0.097±0.092) for adjacent normal mucosa. The relative expression of Lgr5 and VEGF protein in cancer was significantly enhanced in gastric cancer tissue (all P=0.001) compared with normal mucosal tissue. There was a positive correlation between Lgr5 and VEGF protein in gastric cancer (r=0.921, P=0.001) (Figure 2D).

### siRNA suppressed Lgr5 expression and affected the expression level of VEGF in gastric cancer cells

**Figure 3:**
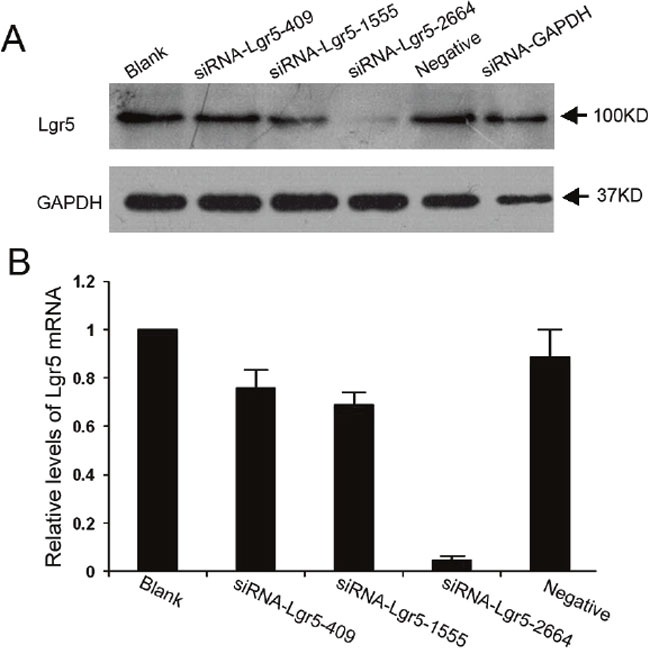
Suppression of Lgr5 protein and mRNA expression by siRNA in AGS gastric cells **A** and **B**. The effect of different siRNAs on the expression of Lgr5 mRNA and protein. Gastric cancer AGS cells were seeded into 6-well plates and transfected with three siRNAs targeting Lgr5 (siRNA-Lgr5-409, siRNA-Lgr5-1555, and siRNA-Lgr5-2664) or scramble siRNA (negative control). The untransfected cells served as a blank control. Lgr5 protein and mRNA levels were detected by western blotting and qRT-PCR. Expression levels of Lgr5 mRNA are presented as mean ± SD. GAPDH was used as an internal control. Triplicate experiments were performed with almost identical results.

Three siRNA duplexes targeting different encoding regions of Lgr5 mRNA, named as Lgr5-homo-409, Lgr5-homo-1555 and Lgr5-homo-2664, were designed and synthesized. These siRNAs were transiently transfected into the AGS gastric cancer cell line. Western blot and qRT-PCR were used to confirm the efficacy of Lgr5 siRNA for suppression of Lgr5 protein and mRNA. As shown in Figure 3, transfection of these Lgr5 siRNA resulted in a significant decrease in Lgr5 protein and mRNA expression. Lgr5-homo-2664 exerted the most efficiency in suppressing Lgr5 expression. Lgr5-homo-2664 siRNAs were transfected into AGS cells, and the transfectants were then selected for further experiments.

**Figure 4:**
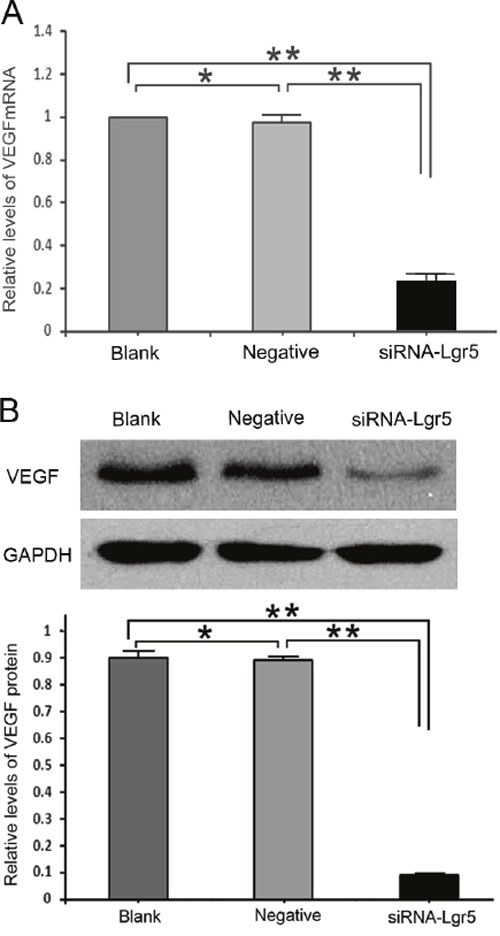
**A**. Lgr5 interference decreased VEGF mRNA and protein expression levels compared with the two control groups (all P = 0.001). **B**. Expression of VEGF in Lgr5-silenced AGS cells and control groups at the protein level using western blotting. Lgr5 interference decreased VEGF protein expression compared with the two control groups (all P = 0.001). The values presented are the mean ± SD from three independent experiments. **P=0.001 compared with cells from the Blank or Negative groups.

The qRT-PCR results indicated that concomitant with Lgr5 downregulation, VEGF mRNA was substantially decreased in Lgr5 siRNA-transfected cells relative to negative-siRNA-transfected cells and untransfected AGS cells (all P=0.001) (Figure 4A). Similar results were observed by western blot analysis (all P=0.001) (Figure 4B). These results demonstrated an important role for Lgr5 in the angiogenesis of gastric cancer.

### Decreased tube formation of HUVECs induced by conditioned medium from Lgr5 siRNA-transfected cells

**Figure 5:**
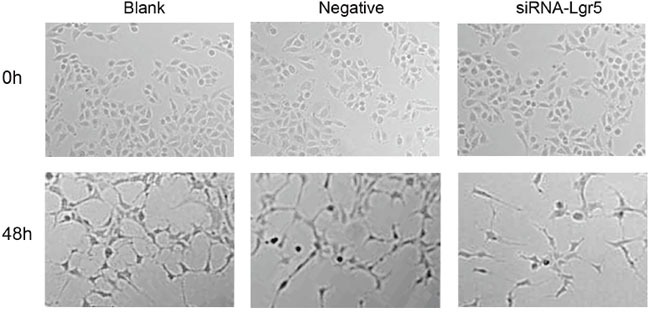
HUVEC were seeded on growth factor-reduced matrigel and stimulated for 48 h with regular cell culture medium (DMEM), conditioned medium from control siRNA-transfected cells, and medium preconditioned with Lgr5 siRNA-transfected cells Tube formation was visualized and calculated by measuring the length of tube walls formed between discrete endothelial cells in each well. In HUVECs stimulated by the medium preconditioned with Lgr5 siRNA-transfected cells, tube formation was significantly suppressed (P=0.001).

We next performed tube formation assays in growth factor-reduced Matrigel *in vitro*. As shown in Figure 5, there were numerous endothelial tube formations after treatment with regular cell culture medium (DMEM) and conditioned medium from control siRNA-transfected cells (7.34 ± 0.30 mm/mm^2^ and 7.18 ± 0.33 mm/mm^2^, respectively). However, when HUVECs were stimulated by the medium preconditioned with Lgr5 siRNA-transfected cells, tube formation was significantly decreased (2.51 ± 0.19 mm/mm^2^) compared with the treatment with DMEM (7.34 ± 0.30 mm/mm^2^) or medium from control siRNA-transfected cells (7.18 ± 0.33 mm/mm^2^) (all P=0.001).

## DISCUSSION

Lgr5, a member of the GPCR superfamily, is overexpressed in many types of human cancer including esophageal adenocarcinoma [15], gastric cancer [16, 17], and colorectal cancer [18–20]. Previous studies demonstrated that Lgr5-positive cells were mainly located in the invasive tumor front of colorectal cancer [40, 41], and significantly correlated with metastasis in regional lymph nodes, distant metastasis, and pTNM stage [18]. Simon et al. [16] reported that Lgr5 expression correlated significantly with the depth of tumor infiltration, lymph node metastasis, and pTNM stage. All these findings suggested that Lgr5 positive cells play a key role in invasion and metastasis in cancer.

Invasion and metastasis of tumors require induction of angiogenesis [42, 43]. Without angiogenesis, most tumors, including gastric cancer, cannot grow beyond a minimal size [44]. Angiogenesis is an important prerequisite for tumor metastasis by increasing the possibility of tumor cells entering the circulation and providing oxygen and nutrients for the metastatic cancer [45]. Highly vascularized gastric cancers are more likely to have lymph node metastasis and peritoneal dissemination than cancers without high vascularization [46].

Currently, MVD, which can be quantified by the specific marker CD34 for vascular endothelial cells, has been widely used to estimate the degree of tumor angiogenesis [47, 48]. VEGF, one of the most important mediators of tumor angiogenesis, can promote the formation of new blood vessels, prevent the regression of vessels and increase microvascular permeability [28, 49, 50]. However, the effect of Lgr5 on tumor angiogenesis has not been examined. In the present study, we investigated Lgr5 expression in a large sample of gastric cancer tissues, and for the first time showed that Lgr5 expression was significantly and positively correlated with VEGF expression and MVD. These results suggest that Lgr5 may contribute to tumor angiogenesis.

Notably, silencing of Lgr5 expression resulted in suppression of VEGF mRNA and protein. This might relate to the canonical Wnt/β-catenin signaling pathway, a crucial component of vascular development and pathology [28]. Lgr5, a part of the Wnt signaling complex at the membrane level, could specifically recruit the LRPs–Frizzled receptor complex. When Lgr5 is activated by its ligand RSPO1, it could lead to phosphorylation of LRP, and eventually interfere with degradation of the crucial signaling molecule β-catenin. Accumulated β-catenin could then translocate to the nucleus together with the Tcf/Lef family of transcription factors, and regulate the expression of a wide range of target genes [43], including VEGF. Because of that there were seven β-catenin/TCF binding site in the promoter of VEGF, and elevated β-catenin levels could dramatically up-regulate levels of VEGF mRNA and protein. Therefore, we considered that Lgr5 participated in gastric cancer angiogenesis by enhancing Wnt/β-catenin signaling.

Downregulation of Lgr5 expression in gastric cancer cells by the siRNA approach would result in a partial reduction in tumor angiogenesis. In this study, the influence of different types of conditioned medium on the formation of HUVEC tubular structures were investigated by means of construction of two-dimensional gel angiogenesis model *in vitro* using matrix. This model has been proven to be one of the reliable methods for *in vitro* analysis of angiogenesis [51, 52]. Our study showed that when HUVECs were stimulated by the medium preconditioned with Lgr5 siRNA-transfected cells, tube formation was significantly decreased compared with the treatment with DMEM or medium from control siRNA-transfected cells.

This is mainly related to the composition of the conditioned medium. Silencing the expression of Lgr5 can down-regulate the expression of VEGF, resulting in a reduced of VEGF protein secretion to the extracellular, which leads to the decrease of VEGF levels in the conditioned medium preconditioned with Lgr5 siRNA-transfected cells. In general, VEGF activates a signaling pathway downstream of the blood vessel by combining with the VEGFR on the vascular endothelial cell membrane to promote angiogenesis. Decreased expression of VEGF could affect the activity of the signaling pathway, thus affecting the angiogenesis. Our results indicate that Lgr5-specific siRNA could be developed as an effective therapeutic agent for patients with Lgr5 overexpressing gastric cancer. Liu et al. demonstrated that the intratumoral injection of siRNAs, which is considered a feasible and convenient method, can be taken as a valuable new approach for the treatment of human cancer [53]. Thus further *in vivo* studies are required to better assess the effectiveness of Lgr5-specific siRNA in inhibiting angiogenesis of gastric cancer.

In conclusion, our results show that Lgr5 is commonly upregulated in human gastric cancer. High Lgr5 expression was also associated with gastric cancer angiogenesis. Silencing the expression of Lgr5 could efficiently inhibit the angiogenesis of gastric cancer at least partially through its suppression effects on VEGF expression. Therefore, our study suggests that Lgr5-specific siRNA could be designed into an effective therapeutic agent to inhibit angiogenesis to achieve the purpose of controlling the growth and metastasis of gastric cancer.

## MATERIALS AND METHODS

### Patients and specimens

A total of 318 gastric carcinoma tissue specimens were obtained from gastric cancer patients who underwent gastrectomy at Chinese People's Liberation Army (PLA) General Hospital (Beijing, China) from 1999 to 2004. The 80 distal normal gastric tissues were randomly selected from the 318 cases of gastric cancer as normal controls. This study was conducted with the approval of the Chinese People's Liberation Army General Hospital Research ethics committee. Tissues were fixed in formalin-fixed and embedded in paraffin. These specimens were used to detect Lgr5, VEGF, and CD34 expression by immunohistochemical staining.

A series of 75 paired fresh cancerous and matched adjacent normal mucosal tissues were collected from gastric cancer patients who were undergoing gastrectomy at the PLA General Hospital in 2010; the sample were snap-frozen at −80°C until the protein extraction was performed.

### Ethical approval

The study with human samples was approved by the Medical Ethic Committee of the Chinese People's Liberation Army General Hospital and was all procedures performed in studies involving human samples were in accordance with the ethical standards of the Medical Ethic Committee of the Chinese People's Liberation Army General Hospital. Written informed consents were obtained from all patients before operation.

### Immunohistochemistry

Immunohistochemical staining of Lgr5, VEGF, and CD34 was carried out according to the procedure previously described [54]. Sections were cut 4 μm thick from formalin-fixed, paraffin-embedded tissue and deparaffinized in xylene and rehydrated. Slides were heated in 0.01 mol/L citrate buffer (pH 6.0) in a microwave oven for 2 min and 30 s at 100°C for antigen retrieval. Then the slides were immersed in 3% hydrogen peroxidase-methanol to inhibit endogenous peroxidase activity. After washing with phosphate-buffered saline (PBS), and blocking with 10% goat serum, the sections were incubated with primary monoclonal rabbit antibody to human Lgr5 (Abcam, Cambridge, Massachusetts, USA) diluted 1:50 in blocking solution, polyclonal rabbit antibody to human VEGF (Santa Cruz, California, USA) diluted 1:250 in blocking solution and mouse monoclonal antibody to human CD34 (Santa Cruz, California, USA) diluted 1:20 in blocking solution. Sections were incubated at overnight 4°C. The sections were rinsed in PBS and incubated with biotinylated secondary antibody (polyperoxidase-anti-mouse/rabbit IgG, Zymed Laboratories Inc., South San Francisco, CA) for 30 min. After washing in PBS, peroxidase reactivity was visualized using a 3,3′-Diaminobenzidine (DAB) substrate kit (Zymed). Slides were counterstained with hematoxylin. The primary antibody was replaced by PBS as a negative control.

### Evaluation of immunohistochemistry and MVD

Antigen immunostaining was evaluated on whole standard tissue sections of gastric carcinoma by two investigators independently who were blind to all clinical data, using a light microscope. For discrepancy cases, a final score was established by re-assessment on a double-headed microscope. In scoring expression of Lgr5 and VEGF protein, both the extent and intensity of immunopositivity were considered, according to Zhao et al. [55] and Hao et al [56]. The intensity of staining was divided into four categories: 0, no staining; 1, weak staining; 2, moderate staining; and 3, strong staining. The proportion of positive cells was divided into four groups: 0, no staining; 1+, positive staining in <10% of the cells; 2+, positive staining covering 10–50%; and 3+, >50% stained positive. The final score was determined by the combined staining score. A score (extent + intensity) ≤ 1 was considered negative, and a score between 2 and 6 was considered positive [57, 58].

The microvessel detection and counting was carried out according to the method as previously described [59]. Briefly, immunohistochemical staining of CD34 was used for MVD counting. The generally accepted criteria for determining a vessel profile was used [60, 61]. Any brown stained endothelial cell or groups of endothelial cell clearly separated from the adjacent microvesssel, tumor cells, and other connective tissue element were considered to be quantifiable individual vessels. The ramified structures were quantified as a single vessel. The entire stained tumor sections were scanned at low magnification (×40) under a light microscope to find five regions with most intense neovascularization. Vessels were counted in each region at high magnification (×200). The counts were performed independently by two investigators, and the mean value was used for analysis.

### Protein extraction and western blotting

Frozen gastric cancer tissues or cells were prepared with lysis buffer. The lysates were harvested by centrifugation (12,000 rpm) at 4°C for 30 min. The protein concentration was determined by Bradford method (Bio-Rad, Hercules, CA). Equal amounts of protein (50 μg/lane) were separated by electrophoresis in sodium dodecyl sulfate polyacrylamide gel (SDS-PAGE), and transferred onto a nitrocellulose membrane (Amersham Biosciences). After blocking with 5% non-fat milk in TBST (50 mmol/L Tris–HCl [pH 7.6], 150 mmol/L NaCl, 0.1% Tween 20) at room temperature for 1 h, the membranes were incubated overnight at 4°C with primary antibody (anti-Lgr5, 1:100, Abcam, Cambridge, Massachusetts, USA; anti-VEGF, 1:400, Santa Cruz Biotechnology, USA; β-actin, 1:1000, Santa Cruz Biotechnology, USA). After washing three times with TBST, the membranes were incubated with horseradish peroxidase-coupled goat anti-rabbit secondary antibody (1:2000, Santa Cruz Biotechnology, USA) for 2 h at room temperature. Antibodies against β-actin and GAPDH were used as internal controls. Enhanced chemoluminescence was used for detection. The protein quantity was analyzed with Quantity-One v4.4 software (Bio-Rad, Hercules, CA, USA). The target protein expression was evaluated by the relative intensity ratio of target protein/loading control.

### Cell Culture

The human gastric cancer cell line AGS and human umbilical vein endothelial cells (HUVECs) were purchased from the American Tissue Culture Collection (ATCC, Manassas, VA, USA). AGS cells were cultured in RPMI 1640 medium (GIBCO-BRL, Gaithesberg, MD) supplemented with 10% fetal bovine serum (FBS, GIBCO-BRL), 100 U/mL penicillin and 100 μg/mL streptomycin. HUVECs (ATCC) were cultured in F12K medium (ATCC) supplemented with 10% FBS (GIBCO-BRL), 0.1 mg/mL heparin sulfate, 0.05 mg/mL endothelial cell growth factor supplement (BD Bioscience, USA), 100 U/mL penicillin, and 100 μg/mL streptomycin. All cells were cultured in a 5% at 37°C in a humidified atmosphere of 5% CO_2_.

### Capillary tube formation assay

The Lgr5 siRNA-or negative-siRNA-transfected control group were cultured in serum-free RPMI 1640 for 72 h. The conditioned media were separately collected, centrifuged and stored at −20°C until use. Regular cell culture medium (DMEM) was used for blank control. Growth factor-reduced Matrigel Matrix (Becton Dickinson, USA) (100 μL) was added to wells of 24-well plates and allowed to polymerize at 37°C for 30 min. HUVECs (1×10^5^ cell per well) in 500 μL of the indicated conditioned medium were seeded in 24-well plate. The chamber was incubated at 37°C in a humidified atmosphere of 95% air and 5% CO_2_. All assays were performed in n = 6/group. For quantitative measurements of the HUVEC tube formation, each well was visualized and digitized under an Olympus inverted microscope with a digital camera. The total tube formation was visualized and calculated by measuring the length of tube walls formed between discrete endothelial cells by Image-Pro Plus 7.0 software in each field of view. The tube formation index was expressed as tube length (mm) per mm^2^ area [62].

### Quantitative real-time, reverse transcription polymerase chain reaction (qRT-PCR)

Total RNA was isolated from cells using an RNeasy Mini Kit (Qiagen, Tokyo, Japan) and reverse transcribed using a cDNA Reverse Transcription Kit (Applied Biosystems, Foster City, CA, USA) according to the manufacturer's instructions. Quantitative real time PCR (QRT-PCR) analysis was performed on an ABI PRISM 7700 sequence detection system (Applied Biosystems) using SYBR GreenPCR Master Mix (Applied Biosystems) at 95°C for 10 min, followed by 50 cycles of 95°C for 15 s and 60°C for 1 min. Three replicates of each sample were analyzed. Primer sequences were as follows: Lgr5 primer (161 bp), forward 5′-TTTGGACAAGGGAGACCTGGAGAAT-3′, reverse 5′-GAAAGCCACAGGGCAGTTTAGGAT-3′; VEGF primer (123 bp), forward 5′-CTTGCCTT GCTGCTCTACCT-3′, reverse 5′-GCAGTAGCTGC GCTGATAGA-3′; and GAPDH primer (266 bp), forward 5′-AGAAGGCTGGGGCTCATTTG-3′, reverse 5′-AGGGGCCATCCACAGTCTTC-3′. Relative values of transcripts were calculated using the 2^−ΔΔC(T)^ method [63]. The mRNA expression level of Lgr5 was normalized to that of GAPDH.

### Transient transfection of Lgr5 siRNA

Lgr5 siRNAs were synthesized by GenePharma Company (Shanghai, China). The sequences were as follows, Lgr5-homo-409: 5′-GCAGAAU AAUCAGCUAAGATT-3′ (sense), and 5′-UCUUAG CUGAUUAUUCUGCTT-3′ (antisense);

Lgr5-homo-1555: 5′-GGACGACCUUCAUAAGA AATT-3′ (sense), and 5′-UUUCUUAUGAAGGU CGUCCTT-3′ (antisense);

Lgr5-homo-2664: 5′-GCUCCAGCAUCACUUA UGATT-3′ (sense), and 5′-UCAUAAGUGAUGCUGG AGCTT-3′ (antisense);

Negative control: 5′-UUCUCCGAACGUGUCA CGUTT-3′ (sense), and 5′-ACGUGACACGUUCGG AGAATT-3′ (antisense);

GAPDH positive control: 5′-GUAUGACAAC AGCCUCAAGTT-3′ (sense), and 5′-CUUGAGGCU GUUGUCAUACTT-3′ (antisense).

AGS cells overexpressing Lgr5 were cultured in 6-well plates at a density of 5 × 10^5^/ml, and then were transiently transfected with 4 μL of siRNA using 2 μl of Lipofectamine 2000 (Invitrogen, USA), according to the manufacturer's instructions.

### Statistical analysis

SPSS V.13.0 (SPSS, Chicago, IL, USA) was used for the statistical analysis. Pearson The Spearman's correlation coefficient test was used to assess the association between expression of Lgr5 and VEGF, and MVD. All quantitative data were presented as the mean ± SD. The Pearson's correlation was used to assess the association between Lgr5 and VEGF relative expression. Differences of the variables between groups were analyzed by the Student's t-test. A value of P<0.05 was considered statistically signifcant.
